# Delayed persistence of elevated monocytic MDSC associates with deleterious outcomes in septic shock: a retrospective cohort study

**DOI:** 10.1186/s13054-020-02857-y

**Published:** 2020-04-07

**Authors:** Louis Waeckel, Fabienne Venet, Morgane Gossez, Céline Monard, Thomas Rimmelé, Guillaume Monneret

**Affiliations:** 1 Immunology Laboratory, Edouard Herriot Hospital, Hospices Civils de Lyon, Pavillon E - 5 place d’Arsonval, 69437 Lyon Cedex 03, France; 2 EA 7426 “Pathophysiology of Injury-Induced Immunosuppression” (Université Claude Bernard Lyon 1 - Hospices Civils de Lyon - bioMérieux), Edouard Herriot Hospital, 69437 Lyon, France; 3 Anesthesia and Critical Care Medicine Department, Edouard Herriot Hospital, Hospices Civils de Lyon, 69437 Lyon, France

**Keywords:** MDSC, Sepsis, HLA-DR, Monocyte, Flow cytometry

To the editor,

Recent observations indicate that some septic patients, after inaugural inflammation, enter a stage of protracted immunosuppression that may take weeks/months to vanish and associate with increased rate of secondary infections and mortality [1]. Delayed elevation of myeloid-derived suppressor cells (MDSC) has recently been hypothesized as a key mechanism sustaining sepsis-induced immunosuppression [2, 3]. These cells constitute a heterogeneous population of immature myeloid cells characterized by their capacity to suppress T cell response [4]. There are 3 major MDSC subsets [5]: granulocytic/neutrophilic MDSC (PMN-MDSC phenotypically and morphologically similar to neutrophils), monocytic MDSC (M-MDSC phenotypically and morphologically similar to monocytes), and early MDSC (eMDSC) which are largely immature and do not express any lineage markers. In humans, M-MDSC are mainly defined as CD14+HLA-DRlow monocytes [5]. In various cancers, these cells revealed as valuable predictive markers of pejorative evolution and as potential targets for innovative therapeutic interventions aimed at abrogating their deleterious immunosuppressive properties. Interestingly, our lab has been focusing for years on the monitoring of the decreased expression of HLA-DR on monocytes (mHLA-DR) in septic shock patients (IMMUNOSEPSIS cohort, #NCT02803346) according to a flow cytometry protocol combining CD14 and HLA-DR detections. Results are expressed as numbers of anti-HLA-DR antibodies bound per monocyte (AB/C) [6]. This parameter has been reported as a reliable predictor of deleterious outcomes after sepsis [1].

According to recommendations for M-MDSC characterization, we developed an automated standardized reanalysis protocol (Altrabio algorithm, www.altrabio.com) to investigate our results under a “MDSC” angle (Fig. 1a). The objective was to assess whether, as in cancer, %M-MDSC may provide relevant clinical information. This percentage was recalculated in 301 patients with septic shock (similar to the recent Sepsis-3 criteria). These patients were enrolled from March 2014 to July 2018 and sampled at days 1–2 and/or days 3–4 and days 6–8 (if still present in ICU) after syndrome onset. Mortality, assessed at day 28, was 34%. Fifty patients (15%) developed ICU-acquired infection (HAI). At days 3–4, these patients presented with usual features of sepsis-induced immunosuppression, i.e., very low mHLA-DR (AB/C), 5150 [3115–8250], and severe lymphopenia: CD4 lymphocyte/μL, 362 [245–591].

**Fig. 1:**
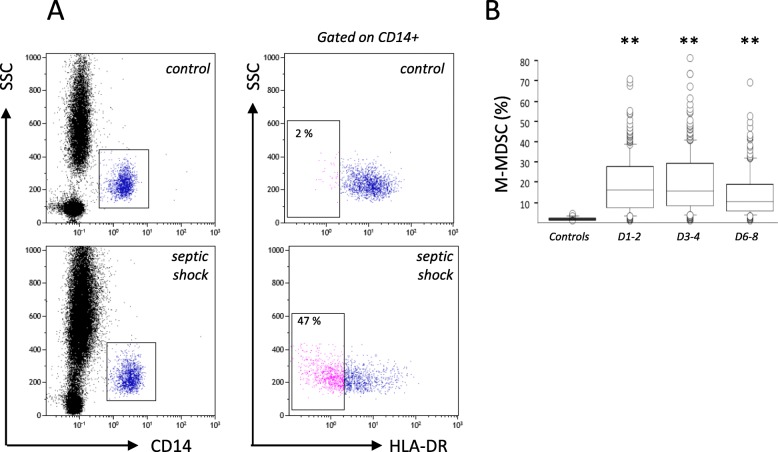
Increased %M-MDSC in septic shock patients. **a** Representative gating strategy used to identify M-MDSC in whole blood from one healthy donor (control) and one patient (septic shock). M-MDSC (%) is the proportion of low HLA-DR monocytes among total CD14+ monocytes. **b** M-MDSC in patients during the first week after septic shock onset (days 1–2, *n* = 259; days 3–4, *n* = 260; and days 6–8, *n* = 168). Patients’ main characteristics were as follows (% or median ± IQR): 67% male; age, 71 [63–79]; SAPS II at inclusion, 60 [49–73]; SOFA at D1, 9 [7–12]; Charlson, 2 [1–4]; and serum lactate at inclusion (mmol/L), 2.7 [1.8–4.4]. Main diagnosis categories were medical (49%)/surgical (51%), types of infection acquisition were community (55%)/nosocomial (45%), and the most frequent sites of infection were abdominal and pulmonary. Missing values corresponded to patients who died or left ICU before days 6–8 and to missing samples during the weekends for which staining was not possible since lab was not operating 24/7. Seventeen healthy donors served as controls (9 women and 8 men, median age was 49, range 28–62). Comparisons (each time point vs controls) based on Mann-Whitney *U* test (**p < 0.01)

We observed a marked elevation of M-MDSC in patients in comparison with healthy controls (*n* = 18, all values < 2%, Fig. 1b). Not surprisingly, there was a strong negative correlation between mHLA-DR and %M-MDSC. Regarding M-MDSC association with deleterious outcomes (i.e., 28-day mortality or HAI), we did not find any difference at the first time points. However, at days 6–8, patients who were going to die or to get infected presented with significantly higher M-MDSC values (Fig. 2a, b). These associations remained significant in multivariate analyses including usual potential confounders (age, gender, SAPS II, SOFA, comorbidities, mechanical ventilation). Calculated odds ratios were 4.4 (*p* = 0.001) and 2.4 (*p* = 0.013) for mortality and nosocomial infection occurrence, respectively. Accordingly, Kaplan-Meier representations clearly illustrate the pejorative evolution of patients with the highest M-MDSC values (Fig. 2a, b).

**Fig. 2:**
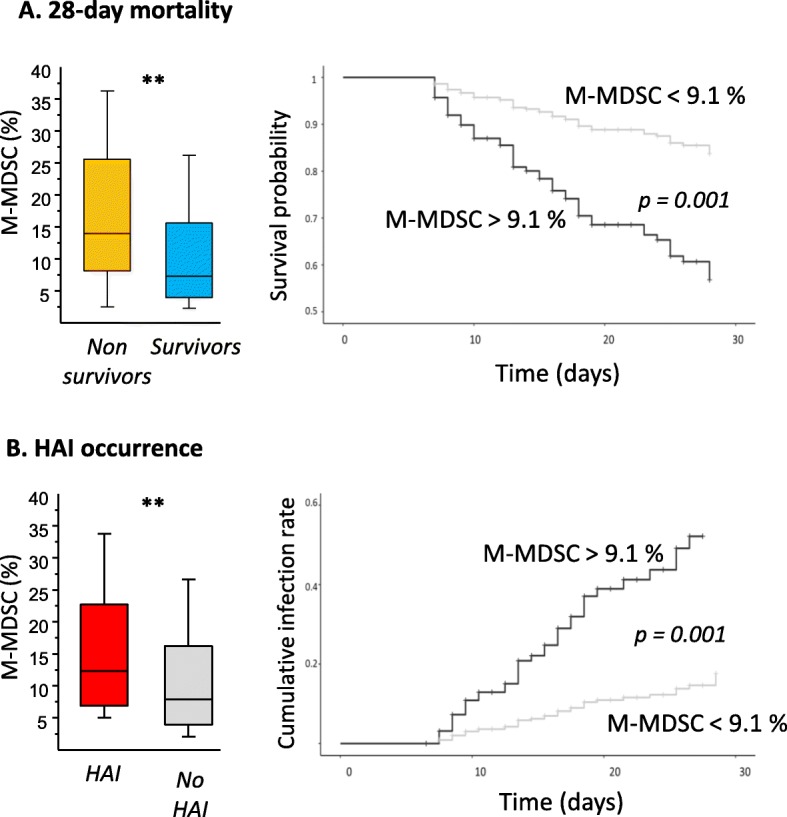
%M-MDSC at days 6–8 associated with clinical outcomes. **a** %M-MDSC among total monocyte population at days 6–8 after septic shock according to survival status at day 28: survivors (*n* = 123) and non-survivors (*n* = 45). Left, comparison between groups: Mann-Whitney (**p < 0.01). Right, Kaplan-Meier survival curves, patients were stratified in 2 groups based on median %M-MDSC value at days 6–8, difference between curves based on log-rank test. **b** %M-MDSC among total monocyte population at days 6–8 after septic shock according to the occurrence of health care-associated infections (HAI): patients developing HAI (*n* = 39) or not (*n* = 129). Left, comparison between groups: Mann-Whitney (**p < 0.01). Right, cumulative incidence curves for HAI stratified based on median %M-MDSC value at days 6–8, difference between curves based on log-rank test

Due to its retrospective nature, the current report presents with limitations. Indeed, while our findings strongly suggest that CD14+HLA-DRlow cells are M-MDSC, definitive proof of their M-MDSC phenotype is presently lacking (i.e., CD15 staining and functional testing). Awaiting confirmation, they may be called “M-MDSC-like cells.”

Overall, the present results provide robust complementary information to that recently published by Hollen and colleagues [3]. Indeed, we observed that the persistence of increased M-MDSC after 1 week was significantly associated with worsening.

## Data Availability

The datasets used and/or analyzed during the current study are available from the corresponding author on reasonable request.

## Abbreviations

HLA-DR: Human leukocyte antigen—DR isotype
MDSC: Myeloid-derived suppressor cells
AB/C: Antibodies bound per monocyte
ICU: Intensive care unit
